# Electrosprayed Particles Loaded with Kartogenin as a Potential Osteochondral Repair Implant

**DOI:** 10.3390/polym15051275

**Published:** 2023-03-02

**Authors:** Sebastian J. Gurgul, Anabela Moreira, Yi Xiao, Swastina Nath Varma, Chaozong Liu, Pedro F. Costa, Gareth R. Williams

**Affiliations:** 1 UCL School of Pharmacy, University College London, London WC1N 1AX, UK; 2 Biofabics, 4200-135 Porto, Portugal; 3 Institute of Orthopaedic and Musculoskeletal Science, University College London, Royal National Orthopaedic Hospital, Stanmore HA7 4AP, UK

**Keywords:** restorative medicine, bone repair, kartogenin, electrospraying, human osteoblasts

## Abstract

The restoration of cartilage damage is a slow and not always successful process. Kartogenin (KGN) has significant potential in this space—it is able to induce the chondrogenic differentiation of stem cells and protect articular chondrocytes. In this work, a series of poly(lactic-co-glycolic acid) (PLGA)-based particles loaded with KGN were successfully electrosprayed. In this family of materials, PLGA was blended with a hydrophilic polymer (either polyethyleneglycol (PEG) or polyvinylpyrrolidone (PVP)) to control the release rate. Spherical particles with sizes in the range of 2.4–4.1 µm were fabricated. They were found to comprise amorphous solid dispersions, with high entrapment efficiencies of >93%. The various blends of polymers had a range of release profiles. The PLGA-KGN particles displayed the slowest release rate, and blending with PVP or PEG led to faster release profiles, with most systems giving a high burst release in the first 24 h. The range of release profiles observed offers the potential to provide a precisely tailored profile via preparing physical mixtures of the materials. The formulations are highly cytocompatible with primary human osteoblasts.

## 1. Introduction

The native osteochondral (OC) unit is a multi-layer gradient system composed of articular cartilage, calcified cartilage, and the underlying subchondral bone. It plays an important role in buffering stress and strain [1]. The articular cartilage is a highly organized tissue that is easily damaged and has limited self-repair capabilities [2]. In normal conditions, articular cartilage has homeostatic crosstalk with bone; however, this homeostasis can be disturbed when damage occurs. Cartilage damage is a major problem, with around 250 million patients suffering from it globally and this number increasing yearly [3]. Cartilage damage can manifest itself as dysregulated bone remodeling, imbalanced cartilage regulation, and progressive OC degeneration caused by trauma or degenerative diseases [4]. Over the past years, many therapies, such as physiotherapy and non-steroidal anti-inflammatory drugs (NSAIDs), have been applied to treat cartilage damage and have proven to relieve the symptoms. However, none of the current treatments can provide complete and long-term repair and restore the function of cartilage tissue [5]. Considering the socioeconomic impact of cartilage damage, improved treatments that have long-lasting therapeutic effects are urgently needed.

One innovative strategy for treating cartilage damage is the approach of tissue engineering, which has been studied by many researchers because of its potential to construct new healthy tissues [6]. There are two requirements for a scaffold used in tissue engineering. Firstly, it must be biocompatible and biodegradable to ensure safety. Secondly, it should be able to mimic the mechanical properties of the native tissue, which means that the scaffold should have a porous structure with properties similar to those of bone tissue [7]. Because of the attractiveness of cartilage tissue engineering, many types of biomaterials have been produced to mimic the structure of the OC unit [8].

Electrohydrodynamic (EHD) techniques use electricity to solidify a solution or melt and have attracted considerable attention for the fabrication of tissue engineering scaffolds. Such formulations can achieve local, sustained, and effective delivery of active ingredients to cells located on the scaffold [9]. Electrospraying is a form of EHD that can generate polymer-based micro- and nanoparticles. It has been explored for several pharmaceutical applications [10,11,12,13,14] and has a number of advantages over other, more conventional, drying techniques such as spray drying or hot melt extrusion. In particular, because electrospraying does not rely on heat to dry, it can be employed to make solid dispersions that contain heat-sensitive drugs. It can also yield smaller particles than other approaches, and since it applies electricity directly to achieve drying (rather than converting electrical to heat energy) it is greener and more energy-efficient than other technologies [15].

The experimental setup for electrospraying consists of four basic components: a syringe fitted with a narrow-bore needle (the spinneret), a pump, a high-voltage power supply, and a collector plate. The pump is used to infuse a polymer solution through the spinneret. The application of an electrical field between this and the collector plate causes the pendant droplet to deform into a conical shape (the Taylor cone), and then it undergoes a Coulombic explosion to generate fine droplets. As these travel toward the collector, they become increasingly small, and the solvent present is evaporated. This results in dry particles being deposited, with sizes on the nm-to-µm scale. The product properties can be tuned by varying the solution properties (polymer concentration, viscosity, conductivity), processing parameters (voltage, flow rate, spinneret-to-collector distance), and environmental conditions (temperature, relative humidity) [16,17].

Several small molecules have been developed using high-throughput screening techniques for the repair of bone defects [18]. The active pharmaceutical ingredient (API) kartogenin (KGN) is one such drug, which can not only reduce cartilage degeneration but also enhance the differentiation of human bone marrow–derived mesenchymal stem cells (hBMSCs) into chondrocytes (CH) [19,20]. This is important as chondrogenesis at the growth plate (epiphyseal plate) drives upgrowth by the elongation of long bones and helps repair cartilage damage. This process results from CH hypertrophy, proliferation, and extracellular matrix secretion. It is prearranged by a complex network of paracrine, cellular, nutritional, and endocrine factors [21,22]. Although KGN is a promising drug for cartilage repair, more work is required to target its delivery. Previous studies have reported that the direct injection of KGN into the joints is ineffective because most of the drug is absorbed by the circulatory system [23,24]. The limited cellular uptake demands repeated injections, thus increasing both pain to patients and the risk of infection. To enhance the efficiency of KGN, a local sustained-release formulation is required.

Electrospraying is widely used in the preparation of microspheres for bone-targeted applications. For instance, hBMSCs were successfully embedded in electrosprayed alginate-gelatine B particles [25]. 17β-estradiol (E2)-loaded poly(lactic-co-glycolic acid) (PLGA) nanoparticles incorporated in hydroxyapatite-chitosan scaffolds were developed by Irmak et al. [26] to induce osteogenic differentiation of adipose-derived stem cells (ADSCs) obtained from a rat model. A formulation of PLGA-based nanoparticles with a poly-aspartic acid (poly-Asp) peptide sequence bound with hydroxyapatite, which acts as a molecular tool in bone-focused applications, was reported by Jiang et al. [27].

PLGA is a synthetic biodegradable polymer that has been approved by the US Food and Drug Administration for pharmaceutical use [28] and employed as a small-molecule drug carrier for decades [29,30]. It can be used to construct porous scaffolds that are able to support OC tissue repair through chondrocyte proliferation [31,32]. PLGA has not only excellent biocompatibility but also degradation rates that can be tuned through the variation of the lactic acid/glycolic acid ratio in the polymer [33]. It can also be surface modified to aid cell adhesion [34,35].

The PLGA-based scaffolds reported in the literature include electrospun nanofibers, electrosprayed micro/nanoparticles, 3D-printed systems, and hydrogels [36]. For instance, Li et al. fabricated PLGA/nanohydroxyapatite electrospun fibers and found that the scaffold can efficiently promote osteoblast cell proliferation [37]. Xu et al. developed a composite pearl/PLGA scaffold using 3D printing and reported that it could satisfy the requirements of OC tissue engineering [38]. In other work, Zhao et al. constructed a PLGA(KGN)/chondrocyte extracellular matrix microsphere scaffold and observed that it could provide sustained KGN release [32]. Another group synthesized a PLGA–poly(ethylene glycol)–PLGA triblock copolymer and used this to prepare a KGN-loaded thermogel for intra-articular injection [39]. This scaffold provided a 3-week sustained release of KGN and showed enhanced cartilage regeneration and the inhibition of joint inflammation in a rabbit OC model. These successful examples confirm that PLGA is a promising material for cartilage repair.

There can, however, be a problem with PLGA formulations in that the hydrophobic nature of the polymer can lead to overly slow drug release [40,41]. This can be ameliorated by mixing in a quantity of a hydrophilic polymer [42]. Poly(ethylene glycol) (PEG) is one such polymer and is widely used for solid dispersion formations. PEG is regarded to be safe, though it should be noted that allergic reactions have been reported in some cases [43,44]. Doping varied amounts of PEG into a PLGA formulation should change the drug release profile and permit fine-tuning. PEG has also been extensively studied for preparing hydrogels for OC tissue engineering [45]. PEG hydrogels can provide a swollen network with properties similar to those of cartilage tissues [46]. An injectable hydrogel consisting of PEG and collagen was formed by Sargeant et al. for tissue regeneration [47]. This composite platform showed excellent biodegradability and the ability to promote the proliferation of fibroblasts. Another example is the simvastatin-loaded PLGA–PEG–PLGA hydrogel successfully prepared by Yan et al. [48]. This material showed thermal sensitivity and an excellent osteogenic effect both in vivo and in vitro.

In this work, blends of hydrophilic polymers with PLGA were prepared to generate electrosprayed systems with tunable KGN release. To do this, either PEG or polyvinylpyrrolidone (PVP, another water-soluble, biocompatible, non-toxic polymer [49] widely used in drug release formulations [50,51,52,53]) was used. A set of PLGA, PVP/PLGA, and PEG/PLGA particles containing different amounts of PVP or PEG were fabricated, and these blends were used to fine-tune the release of KGN.

## 2. Materials and Methods

### 2.1. Materials

Chloroform, dimethyl formamide (DMF), polyvinylpyrrolidone (PVP; average Mn 10 k), poly(ethylene glycol) (PEG; average Mn 35k), poly(D,L-lactide-co-glycolide) (PLGA; 85:15 lactide:glycolide ratio, Mn 50 k–75 k), and phosphate-buffered saline (PBS pH = 7.4) were purchased from Sigma-Aldrich (Gillingham, UK). Dimethyl sulfoxide (DMSO) and methanol were sourced from Fisher Scientific (Loughborough, UK). Kartogenin was purchased from Acros Organics (Loughborough, UK). The RealTime-Glo™ MT Cell Viability Assay was obtained from Promega (Southampton, UK). Primary human osteoblasts (HOB) were purchased from PromoCell (Heidelberg, Germany), and complete osteoblast growth medium was bought from Cell Applications, Inc. (San Diego, CA, USA).

### 2.2. Electrospraying of Drug-Loaded Nanoparticles

For the production of PLGA particles, a PLGA solution at a concentration of 4% *w*/*w* was prepared in chloroform, magnetically stirred until all the polymer was dissolved (at least 3–4 h), and loaded into 5 mL plastic syringes (Terumo, Bagshot, UK). A conductive needle (20G, part number: 7018169; Nordson, Aylesbury, UK) was used as the spinneret. The syringes were then mounted on syringe pumps (KDS-100; Cole-Parmer, St. Neots, UK) and the positive electrode of an HCP35-35,000 high-voltage power supply (FuG Elektronik, Schechen, Germany) was clamped to the spinneret. The particles were collected on an aluminum plate covered with aluminum foil, with electrospraying performed in cone-jet mode. Initially, a preliminary optimization (exploring flow rate and voltage) was performed, after which the following set of parameters was chosen to give PLGA particles with clean spherical morphology: flow rate 0.5 mL/h; voltage 10 kV; tip-collector distance 22 cm. Experiments were performed at ca. 21 °C and 45% relative humidity.

Formulations loaded with KGN were then prepared. KGN was first dissolved in 200 μL of DMF to give a KGN concentration of 50 mg/mL (5% *w*/*v*). Chloroform was then added to give a 3% *w*/*v* KGN solution. Finally, PLGA and PEG or PVP were dissolved into the solvent mixture to give a homogenous solution with a total polymer content of 4% *w*/*w*. To generate particles, the same equipment as above was used, but the flow rate was set to 0.4 mL/h. The voltage and collection distance used were optimized for each formulation and fell in the range of 7.1–12.3 kV and 23–25 cm, respectively. Full details are given in the Supporting Information, Tables S1 and S2. As controls, blank (without KGN) formulations of PLGA/PEG and PLGA/PVP were electrosprayed, with the PLGA/PEG and PLGA/PVP ratios set at 70/30% *w*/*w*.

### 2.3. Material Characterization

A MiniFlex 600 instrument (Rigaku, Tokyo, Japan) was used to collect X-ray diffraction (XRD) data. The instrument is equipped with a Cu-Kα radiation source. Raw materials and formulations were loaded into low background glass holders and analyzed in the 2θ range between 2.5° and 50.0°. The step size was 0.02°, and the scan rate was 5.0°/min. Fourier transform infrared (FTIR) spectroscopy was undertaken on a Spectrum 100 spectrometer (PerkinElmer, Waltham, MA, USA). Experiments were performed in attenuated total reflectance mode over the wavenumber range of 4000–600 cm^−1^, with 8 scans recorded at a resolution of 4 cm^−1^. Differential scanning calorimetry (DSC) data were collected on a Q2000 instrument (TA Instruments, New Castle, DE, USA). Next, 3–10 mg of the sample was loaded into a Tzero hermetic pan. The pans were sealed with a Tzero lid and pinholed. Initially, the raw materials and formulations were equilibrated at 20 °C for 5 min and then heated to 300 °C at a rate of 10 °C/min. This procedure was carried out under a nitrogen atmosphere with a flow rate of 50 mL/min. Scanning electron microscopy was undertaken on a Quanta FEG 200 instrument (FEI, Hillsboro, OR, USA) after sputter-coating samples with gold. The size of the particles was calculated using the ImageJ software. At least 30 individual particles were measured for each image.

### 2.4. Encapsulation Efficiency

KGN encapsulation efficiency (EE%) was calculated using Equation (1) [54]:

EE% = (100 × mass of KGN in formulation)/(mass of KGN in feedstock)(1)

To determine the EE%, 5 mg of KGN was dissolved in 1 mL of DMF. This stock solution was then diluted, and a calibration curve was collected on a UV-Vis spectrometer (7315 spectrometer; Jenway, London, UK; 274 nm) with samples mounted in quartz cuvettes (Fisher Scientific, Loughborough, UK); see Supporting Information, Figure S1. Next, 8.75 ± 0.20 mg of each sample was mixed with 1 mL of DMF to give a final drug concentration of ca. 0.25 mg/mL. The solutions were stirred overnight to ensure complete dissolution. The EE% determinations were performed in triplicate and are reported as mean ± standard deviation (S.D.).

The drug loading (DL%) was also calculated from these experiments using Equation (2) [55].

DL% = (100 × mass of KGN in formulation)/(mass of formulation)(2)

### 2.5. Drug Release Studies

A total of 10.5 ± 0.1 mg of each electrosprayed sample was dispersed in a glass vial with 3 mL of PBS (pH 7.4). Considering that the solubility of KGN in an aqueous environment is very limited, the maximum concentration of KGN in each solution was about 0.1 mg/mL (solubility of KGN in water is below 1 mg/mL) [56]. The release study was conducted at 37 °C in an incubator with shaking (150 RPM). Next, 1 mL aliquots were removed at periodic time points, and a constant volume was maintained by adding 1 mL of fresh pre-warmed PBS. The aliquots were subject to centrifugation (15,000 rpm, 10 min), and the concentration of KGN was determined by UV spectroscopy as above, using a predetermined calibration curve (Figure S2).

### 2.6. In Vitro Cell Assay

Primary human osteoblasts (HOB) were used in this study to evaluate the cytotoxicity of KGN and the formulations. Cells were routinely maintained as monolayers at 37 °C in standard cell culture conditions (5% CO_2_/air and 95% humidity). As a control, a series of different concentrations of KGN in DMSO/media were prepared. The concentrations were 0.005, 0.01, and 0.02 mg of KGN per 100 μL of media. The medium employed was a complete osteoblast growth medium, which was used as supplied. Then, 10,000 HOB cells (passage number 7–10) in 100 μL of media were seeded in each well of 96 well plates. Each formulation was added to the cells at a concentration of 0.35 mg per 100 μL, giving 0.01 mg of KGN per 100 μL of media. Three independent experiments were performed with triplicate wells in each plate. The RealTime-Glo MT cell viability assay (Promega, Southampton, UK) was employed to determine viability according to the manufacturer’s instructions. Luminescence was measured on a GloMax^®^ Navigator luminometer (Promega, Southampton, UK).

## 3. Results

### 3.1. SEM Examination

**Figure 1:**
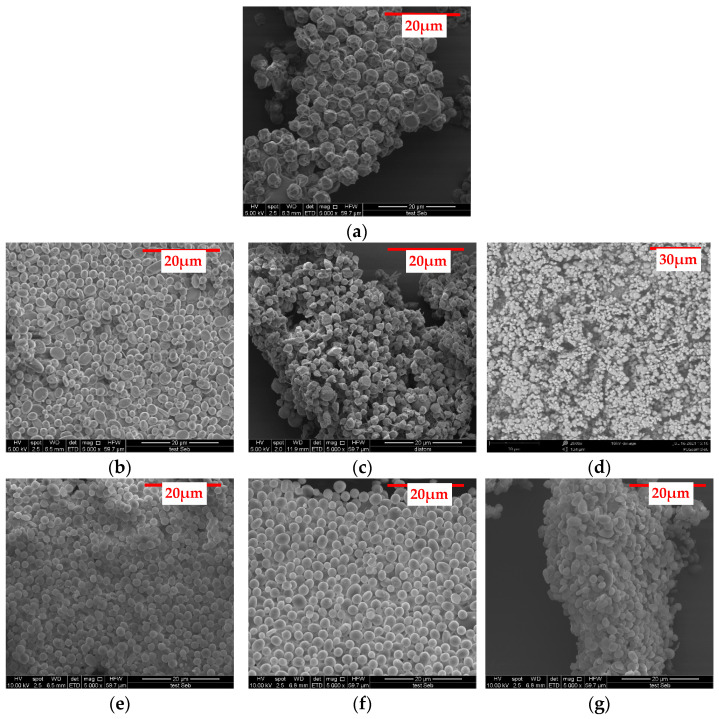
SEM images of KGN-loaded particles prepared with (**a**) PLGA and PLGA combined with (**b**) PEG at 10% *w*/*w*; (**c**) PEG at 30% *w*/*w*; (**d**) PEG at 50% *w*/*w*; (**e**) PVP at 10% *w*/*w*; (**f**) PVP at 30% *w*/*w*; and (**g**) PVP at 50% *w*/*w*. Scale bar: 20 µm for all panels except (**d**) 30 µm.

SEM images of blank PLGA, PLGA/PEG 30% *w*/*w,* and PLGA/PVP 30% *w*/*w* particles are given in Figure S3. The particles were generally spherical in shape and uniform in size. Images of particles formulated from a polymer matrix (PLGA, PLGA/PEG and PLGA/PVP) and loaded with KGN are shown in Figure 1. It was observed that the particles were uniform across the different blends, with spherical morphologies. The particles were always heavily aggregated, and those prepared from PLGA and PLGA/PEG 30% *w*/*w* were indented in places, while the others had smooth surfaces.

The particle sizes were calculated as 3.53 ± 0.93 μm (PLGA), 2.67 ± 0.65 μm (PEG 10% *w*/*w*), 2.9 ± 1.63 μm (PEG 30% *w*/*w*), 2.41 ± 0.81 μm (PEG 50% *w*/*w*), 4.08 ± 1.61 μm (PVP 10% *w*/*w*), 2.56 ± 0.62 μm (PVP 30% *w*/*w*), and 2.77 ± 0.83 μm (PVP 50% *w*/*w*). These sizes were largely consistent, and there were no clear trends to be observed in the data. The particle size is similar to that reported in the literature. For example, Kibler et al. [57] obtained blank particles of diameter 1.23 ± 0.45 μm and around 2.46 ± 0.9 μm in length, and Tanaka et al. [58] prepared 1.30–6.19 μm PLGA particles.

### 3.2. XRD

**Figure 2:**
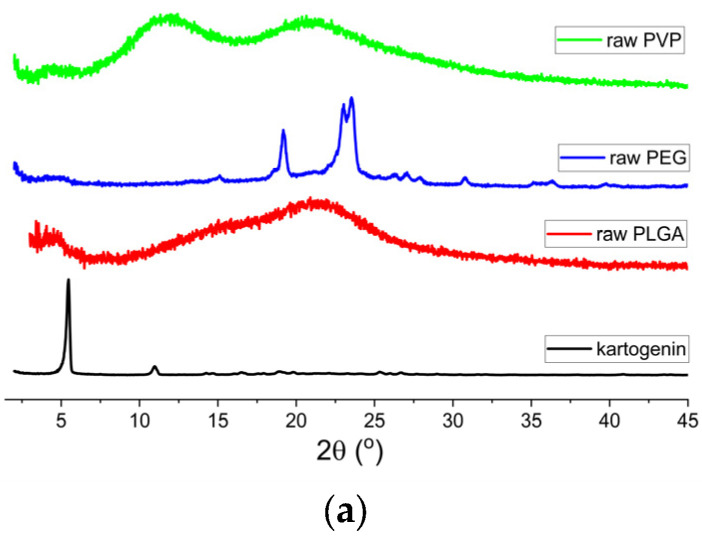

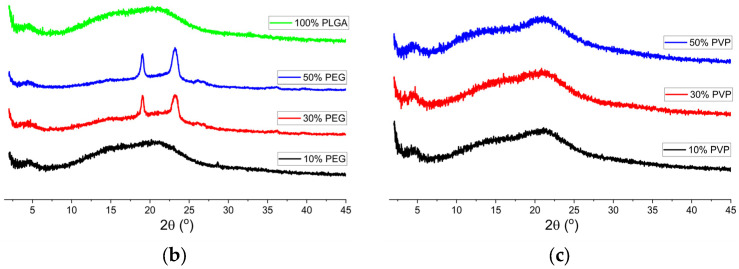
XRD patterns of (**a**) raw materials (PLGA, PEG, PVP, and KGN); (**b**) PLGA and PLGA/PEG particles loaded with KGN; and (**c**) PLGA/PVP particles loaded with KGN.

XRD patterns are depicted in Figure 2.

The XRD pattern of KGN (Figure 2a) shows strong Bragg reflections, indicative of a crystalline material and consistent with the literature [59]. The polymer data (Figure 2a) reveal that PVP and PLGA were both amorphous, with broad halos in their patterns. In contrast, PEG was semi-crystalline, with particularly distinct reflections noted at 20 and 25°. In Figure 2b, the electrosprayed PLGA and PEG/PLGA formulations loaded with KGN are depicted. The KGN-PLGA data (Figure 2b) indicate the formulation was amorphous, and the pattern observed is the same as for raw PLGA (Figure 2a). No peaks for KGN were observed in the formulation, suggesting it was present as a molecular dispersion in the polymer carrier (though it should be noted this could be due to the low drug loading in the formulations making crystalline material hard to detect by XRD). The same is noted for the 10% *w*/*w* PEG formulation. The materials containing higher amounts of PEG showed some Bragg reflections attributable to PEG, but the KGN reflections were absent. Figure 2c indicates that all formulations containing PVP were amorphous.

### 3.3. DSC Analysis

**Figure 3:**
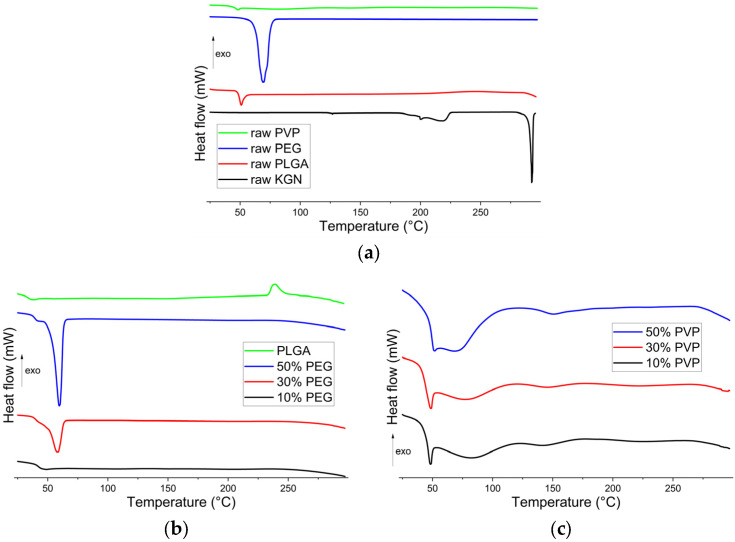
DSC patterns of (**a**) the raw materials (PLGA, PEG, PVP, and KGN); (**b**) KGN-PLGA and KGN-PLGA/PEG particles; and (**c**) KGN-PLGA/PVP particles.

The DSC trace of KGN shows a broad endothermic peak over the range of 180–220 °C (Figure 3a) [59] and a sharp endothermic melting peak at 292 °C [60]. The latter confirms the crystalline nature of the material and agrees with the XRD data. There are a superimposed relaxation endotherm and a baseline shift visible in the trace of PLGA at 50 °C, which correspond to the polymer’s glass transition temperature (*T_g_*). This is in agreement with a literature report that the *T_g_* of PLGA (85/15) is 50.4 ± 0.2 °C [61]. PEG displays a melting endotherm at 68 °C, consistent with its semi-crystalline nature (Figure 3a) [62,63,64]. The DSC trace for PVP is largely featureless except for an event at around 50 °C, which represents water loss upon heating [65]. Again, this is consistent with an amorphous material.

Considering the DSC data for the formulations (Figure 3b,c), the KGN melting endotherm is not visible in any case, which indicates that KGN is present in the amorphous form in all the electrosprayed samples (though, as noted above, the low drug loading means caution must be taken here). It was observed in all cases that the thermograms of the electrosprayed particles resembled a composite of those of their polymer constituents. The PLGA *T_g_* is clearly visible for the PEG and PVP-containing formulations, though it overlaps with the PEG melt or PVP dehydration endotherm in the 30 and 50% *w*/*w* systems. In the PEG formulations (Figure 3b), the PEG melting endotherm (around 58 °C) becomes more intense with an increased concentration of PEG. There is no clear relaxation endotherm from PLGA visible, though there is a shoulder on the PEG melting endotherm for the 30 and 50% PEG systems, which may have arisen from PLGA relaxation. For the PVP-loaded systems (Figure 3c), an increase in PVP concentration manifests itself in a larger dehydration endotherm at 50–100 °C, which is overlaid with the PLGA relaxation endotherm.

### 3.4. FTIR

**Figure 4:**
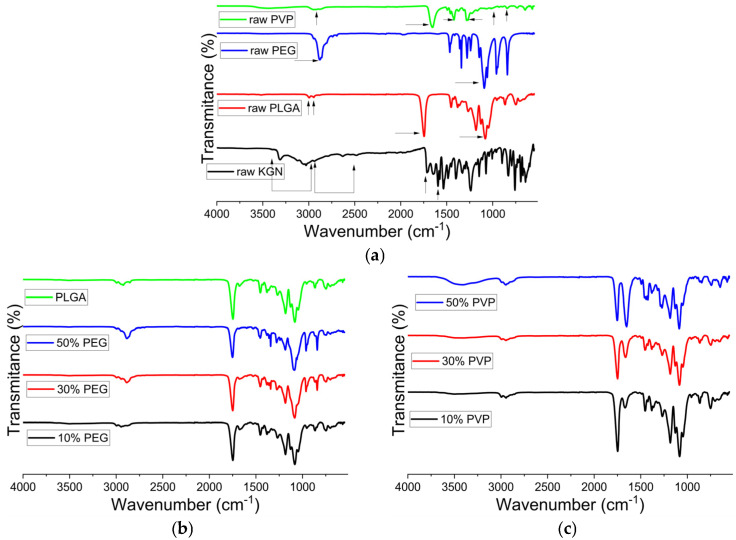
FTIR spectra of (**a**) the raw materials; (**b**) KGN-loaded PLGA and PLGA/PEG particles; and (**c**) KGN-loaded PLGA/PVP particles. The arrows represent the main bands arising in the raw materials.

The FTIR data for the raw materials and KGN-loaded particles are presented in Figure 4.

In Figure 4a, PLGA displays characteristic bands at 3040–2880 cm^−1^ associated with C-H stretching, 1760 cm^−1^ (C=O stretching), and 1184 cm^−1^ (C-O stretching of aliphatic ester). In the PEG spectrum (Figure 4a), C-H stretching appears at 2870 cm^−1^, and another characteristic peak at 1124 cm^−1^ is correlated with C-O stretching (aliphatic ether). PVP has notable bands at 2950 and 1652 cm^−1^ (asymmetric stretching of CH_2_ and stretching of C=O, respectively) and at 1423 and 1288 cm^−1^ (C-H bending and CH2 wagging). The peak at 1018 cm^−1^ is the CH_2_ rocking. KGN has a visible band at 3400–3000 cm^−1^ (N-H stretching), 3000–2500 cm^−1^ (C-H stretching), 1760 cm^−1^ (carboxylic acid C=O stretching), and 1680 cm^−1^ (amide C=O stretching).

The spectra of the KGN-PLGA formulations (Figure 4b) resemble those of PLGA, but no bands from the KGN are visible. The CH_2_ peaks of PEG at 2884 cm^−1^ and 2857 cm^−1^ are still visible in the PLGA/PEG formulations, and it can be noted that a higher content of PEG leads to greater absorption at these positions. The C=O bond of PLGA at 1747 cm^−1^ still exists, but the peak of KGN at 1715 cm^−1^ is not visible. This is because the C=O band of PLGA is dominant over that of KGN. In Figure 4c, the C=O peak of PVP appears as a shoulder on the right of the PLGA C=O peak in the 10% *w*/*w* and 30% *w*/*w* PVP samples, while in the 50% *w*/*w* PVP sample, these two peaks show similar intensities. O-H stretching peaks at 3200 cm^−1^, due to the absorbed water, can be observed in the PVP-containing systems. Again, KGN peaks are not visible. This is likely to be because of the low drug loading in the system.

### 3.5. Encapsulation Efficiency and Drug Loading

The encapsulation efficiencies and drug loadings of KGN in the particles are detailed in Table S2. In general, the encapsulation efficiency was close to 100%, with all values at or above 93 ± 2%. The drug loading was almost the same as the theoretical loading, with a value of 3% *w*/*w* (Table S2) across all formulations.

### 3.6. Release Studies

**Figure 5:**
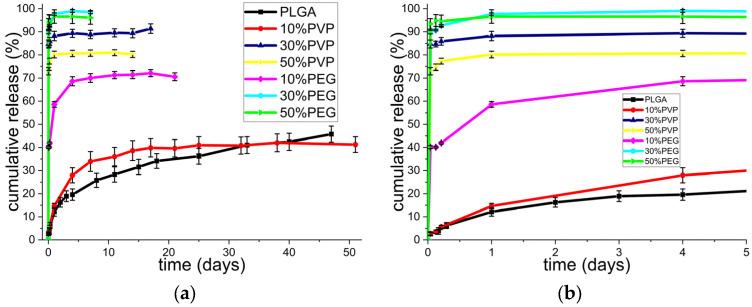
KGN release profiles from PLGA, PLGA/PEG, and PLGA/PVP particles showing (**a**) the full release period and (**b**) an enlargement of the first 5 days. Results are presented as mean ± S.D. from three independent experiments.

The drug release profiles are given in Figure 5.

An initial burst release can be observed in all the samples, though this was notably reduced for PLGA and the 10% *w*/*w* PVP system. The 30% *w*/*w* and 50% *w*/*w* PEG materials had the greatest burst release, with more than 90% of the KGN in the samples released in the first hour, while the 30% *w*/*w* and 50% *w*/*w* PVP systems had a burst release of 80–90%. This phenomenon could be attributed to the fact that a high content of KGN was present on or near the surface of the particle formulations and that the PVP and PEG in the formulation could dissolve into the release medium to create pores. The latter allowed water ingress and for the KGN to escape into the solution. This explanation is in agreement with the findings in the literature [66,67]. The samples with higher PLGA content showed reduced release on the first day, owing to the hydrophobic nature of the polymer. While the PVP and PEG could dissolve rapidly into the solution, the PLGA was slowly eroded. Overall, the best-performing formulation seems to be pure PLGA, as it had almost a zero-order release after the first 5 days. This is followed by the 10% *w*/*w* PVP formulation, which provided sustained release over 3 weeks but then tailed off.

Comparing the scaffolds prepared here with the literature, it can be seen that the KGN release continued from the PLGA formulation over more than 45 days (reaching a little over 40% *w*/*w*), a slower release profile than seen in other studies, which reported 70% release after 35 days when PLGA/KGN particles were fabricated by a single emulsion/solvent evaporation method or simple mixing methods [68,69]. The formulations presented here offer the potential for a wide range of different release profiles through preparing physical mixtures of the different systems. For instance, the PLGA and 30 or 50% *w*/*w* PEG systems could be combined to provide a required loading dose of KGN followed by sustained release.

### 3.7. In Vitro Assay

**Figure 6:**
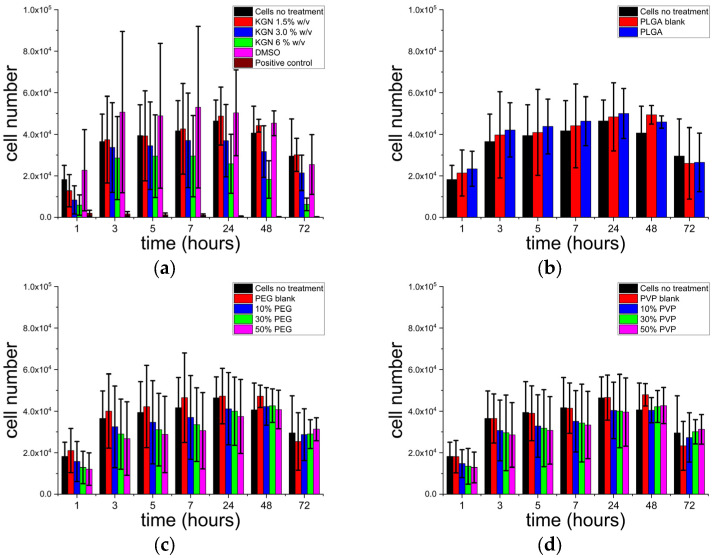
HOB cell numbers for (**a**) different concentrations of KGN; (**b**) PLGA formulations; (**c**) blended PLGA/PEG-KGN formulations; and (**d**) blended PLGA/PVP-KGN formulations. KGN-treated wells were exposed to different concentrations of KGN dissolved in DMSO/culture media, with the final concentration of DMSO being 0.1% *v*/*v*. DMSO control wells were treated with DMSO/media mixture at the same DMSO concentration. ‘Positive control’ are the wells that were treated with 100% ethanol. Statistical analysis using ANOVA revealed no significant differences between the formulations and negative controls.

The viability of HOB cells after exposure to selected formulations is depicted in Figure 6. In Figure 6a, the toxicity of KGN is explored. Cells were treated with a range of concentrations of KGN (1.5, 3, and 6% *w*/*v*, where 3% *w*/*v* represents the same KGN concentration as in the formulations). It is clear that in all cases, the cell viability was very similar to that of the untreated cells and that the presence of KGN in different concentrations was not causing cell death over 72 h. A similar observation was made by Wang et al., where the addition of KGN had no adverse effect on primary human fibroblast viability during 48 h [70].

As can be seen from the data in Figure 6b–d, cell numbers increased up until ca. 24 h, after which they plateaued (48 h) and then declined (72 h). The decline arose because the cells became over-confluent after 48 h (confluence occurs at approximately 40,000 cells per well). The PLGA, PLGA/PEG, and PLGA/PVP formulations did not show any toxicity and, therefore, have the potential to be used as a carrier for KGN. The PLGA, PVP, and PEG blanks indicated that the formulations alone have no negative influence on cell survival and are cytocompatible. Cell proliferation occurred at very similar rates both for the untreated cells control and for the KGN-loaded particles and was not markedly inhibited by the presence of the polymer particles. This is in good agreement with the literature, in which PLGA, PVP, and PEG were all shown to be highly biocompatible [71,72,73,74]. It is also suggestive that along with cell viability, the KGN-loaded formulations do not interfere with cell adhesion as cells continue to proliferate over 72 h.

## 4. Conclusions

In this work, PLGA-based particles with KGN encapsulated were successfully electrosprayed. A series of materials was generated in which PLGA was blended with various ratios of PEG or PVP and KGN. The particles were largely spherical, with relatively uniform sizes in the range of 2.4–4.1 µm. The KGN encapsulation efficiency was high, at >93% of the theoretical loading. XRD and DSC suggested the particles comprised amorphous solid dispersions. KGN features were not clearly visible in the IR data, owing to the low drug loading (3% *w*/*w*). The various materials gave a range of drug release rates, offering the potential for precisely tunable systems. PLGA particles loaded with KGN displayed the slowest release because of the hydrophobicity of PLGA, reaching only 40% release after 45 days. After an initial burst release of ca. 15%, there was almost zero-order release between days 5 and 45. Blending the PLGA with PVP or PEG resulted in markedly changed profiles, with most systems giving a high burst release of >60% in the first 2 h. The exception was the 10% PVP system, which gave a burst release of around 15%. The range of release profiles observed offers the potential to provide a precisely tailored profile, depending on the patient’s needs, by the simple expedient of making physical mixtures of the materials. For instance, a combination of PLGA and PLGA/PEG particles could give a loading dose of KGN (over 1–5 days) followed by sustained release over 45 days or longer. In vitro assay on HOB cells showed that the formulations were cytocompatible and did not affect viability or survival over 72 h. These are promising findings; in the future, we seek to consolidate these by exploring more detailed in vitro experiments (e.g., alkaline phosphatase assay).

## Data Availability

The raw data underpinning this study are available upon request from the authors.

## Supplementary Materials

The following supporting information can be downloaded at: https://www.mdpi.com/article/10.3390/polym15051275/s1, Figure S1: Calibration curve for KGN in DMF; Figure S2: Calibration curve for KGN in PBS; Figure S3: SEM images of blank (a) PLGA; (b) PLGA/PEG 30% *w*/*w*; and (c) PLGA/PVP 30% *w*/*w* particles, with no drug loading; Table S1. Parameters used in the optimized electrospraying processes; Table S2. Composition, encapsulation efficiency and drug loading of the KGN-containing particles (n = 3).
