# Supplementary material for: Electrosprayed Particles Loaded with Kartogenin as a Potential Osteochondral Repair Implant
Source: Polymers (Basel). 2023 Mar 2;15(5):1275. doi: 10.3390/polym15051275 (PMC10007262; doi:10.3390/polym15051275)
Supplement: Supplementary file 1 [file polymers-15-01275-s001.zip › polymers-2201854-supplementary.pdf]

Supplementary Materials for:

Electrosprayed particles loaded with kartogenin as a potential osteochondral repair implant

Sebastian J. Gurgul[a], Anabela Moreira[b], Yi Xiao[a], Swastina Nath Varma[c], Chaozong Liu[c], Pedro Costa[b], Gareth R. Williams[a,*]

[a] UCL School of Pharmacy, University College London, 29-39 Brunswick Square, London WC1N 1AX, UK
[b] Biofabics, R. Alfredo Allen 455, Porto 4200-135, Portugal
[c] Institute of Orthopaedic and Musculoskeletal Science, Royal National Orthopaedic Hospital, University College London, Stanmore HA7 4LP, UK
* Correspondence: g.williams@ucl.ac.uk

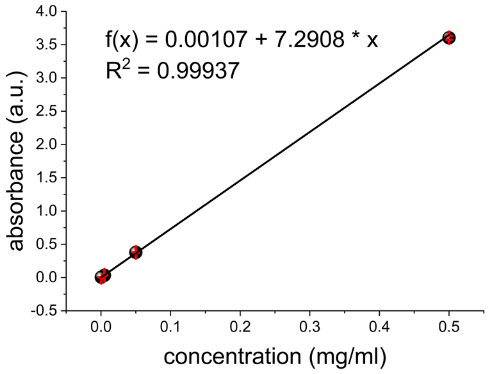

Figure S1. Calibration curve for KGN in DMF.

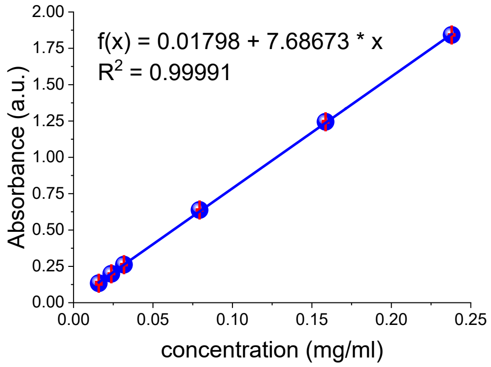

Figure S2. Calibration curve for KGN in PBS.

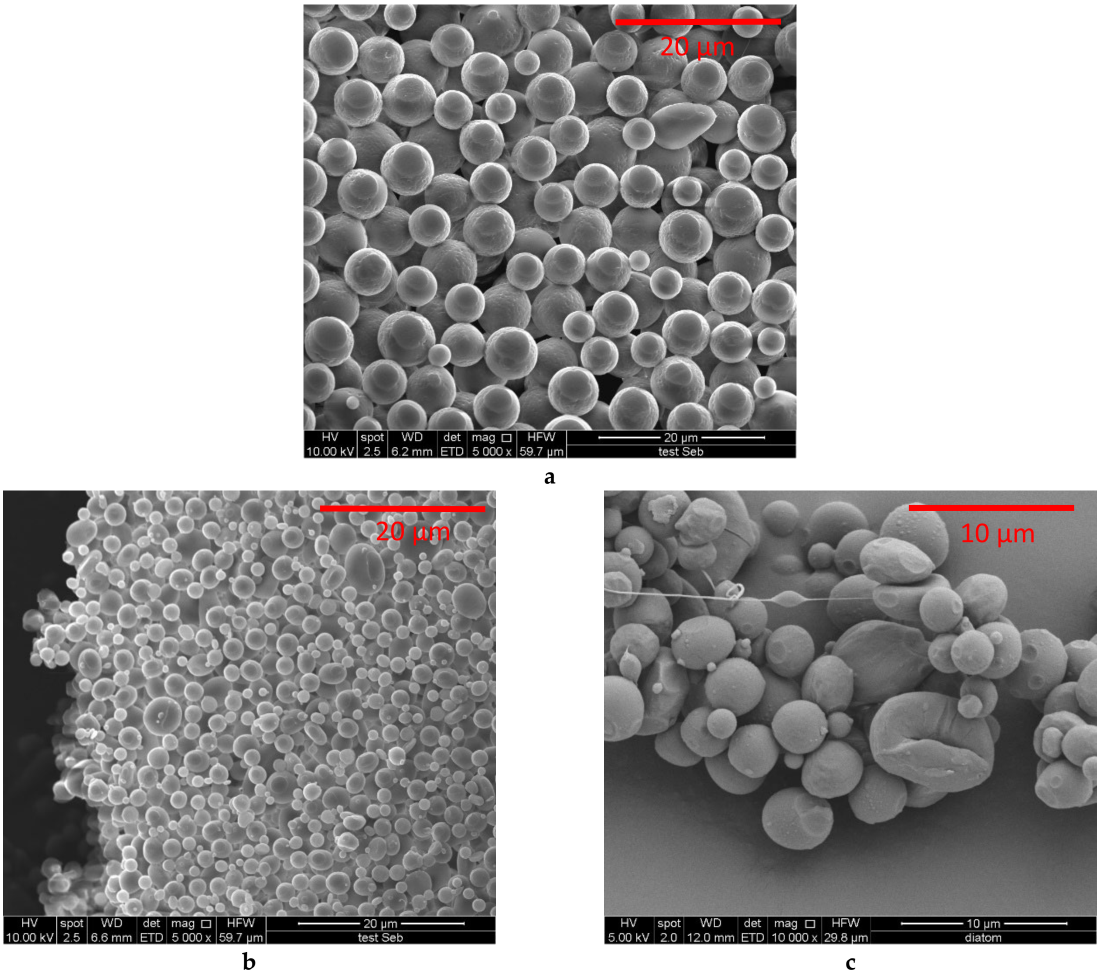

Figure S3. SEM images of blank (a) PLGA; (b) PLGA/PEG 30% w/w; and (c) PLGA/PVP 30% w/w particles, with no drug loading.

Table S1. Parameters used in the optimized electrospraying processes.

**Formulation with Kartogenin**	**Flow Rate (mL/h)**	**Tip-to-Collector Distance (cm)**	**Voltage (kV)**
PLGA	0.4	25	7.0-8.6
10%PEG	0.4	25	8.25-8.4
30%PEG	0.4	24	10.5-11.0
50%PEG	0.4	24	10.5-12.0
10%PVP	0.4	24	7.0-7.8
30%PVP	0.4	24	8.0-10.6
50%PVP	0.4	23	9.2-11.6
**Blank formulation**	**Flow rate (mL/h)**	**Tip-to-collector distance (cm)**	**Voltage (kV)**
PLGA	0.4	25	7.2-7.9
30%PEG	0.4	25	7.1-7.2
30%PVP	0.4	24	7.2-8.4

Table S2. Composition, encapsulation efficiency and drug loading of the KGN-containing particles (n=3).

Sample	PLGA (% w/w)	PEG (% w/w)	PVP (% w/w)	Drug Loading (% w/w)	Encapsulation Efficiency (%)
PLGA	96.8	0	0	3.2 ± 0.2	98 ± 1
10% PEG	87.3	9.7	0	3.0 ± 0.1	98 ± 5
30% PEG	67.9	29.1	0	3.0 ± 0.1	96 ± 1
50% PEG	48.5	48.5	0	3.1 ± 0.0	95 ± 1
10% PVP	87.3	0	9.7	3.0 ± 0.0	94 ± 1
30% PVP	67.9	0	29.1	3.0 ± 0.1	93 ± 2
50% PVP	48.5	0	48.5	2.9 ± 0.1	99 ± 1
